# Cryopreservation of specialized chicken lines using cultured primordial germ cells

**DOI:** 10.3382/ps/pew133

**Published:** 2016-04-20

**Authors:** S. Nandi, J. Whyte, L. Taylor, A. Sherman, V. Nair, P. Kaiser, M. J. McGrew

**Affiliations:** * The Roslin Institute and Royal Dick School of Veterinary Studies, University of Edinburgh, Easter Bush Campus, Midlothian, EH25 9RG, UK; † Avian Oncogenic Virus Group, The Pirbright Institute, Ash Road, Woking, Guildford, Surrey, GU24 0NF, UK

**Keywords:** primordial germ cell, chicken, cryopreservation, biobank, stem cell

## Abstract

Biosecurity and sustainability in poultry production requires reliable germplasm conservation. Germplasm conservation in poultry is more challenging in comparison to other livestock species. Embryo cryopreservation is not feasible for egg-laying animals, and chicken semen conservation has variable success for different chicken breeds. A potential solution is the cryopreservation of the committed diploid stem cell precursors to the gametes, the primordial germ cells (**PGCs**). Primordial germ cells are the lineage-restricted cells found at early embryonic stages in birds and form the sperm and eggs. We demonstrate here, using flocks of partially inbred, lower-fertility, major histocompatibility complex- (**MHC-**) restricted lines of chicken, that we can easily derive and cryopreserve a sufficient number of independent lines of male and female PGCs that would be sufficient to reconstitute a poultry breed. We demonstrate that germ-line transmission can be attained from these PGCs using a commercial layer line of chickens as a surrogate host. This research is a major step in developing and demonstrating that cryopreserved PGCs could be used for the biobanking of specialized flocks of birds used in research settings. The prospective application of this technology to poultry production will further increase sustainability to meet current and future production needs.

## INTRODUCTION

Preservation of genetic diversity of extant populations that can be reintroduced at later times to avoid population bottlenecks is central to controlled flock management. This is especially important for the biosecurity of poultry production systems, which are at risk of emerging disease pandemics such as avian influenza (Whyte et al., in press). There is also a recognized need to preserve and safeguard the genetic diversity of traditional breeds of chicken (Wilkinson et al., 2012). Many of these breeds are maintained in regionally restricted populations and are vulnerable to both disease outbreaks and losses in genetic diversity due to fluctuations in population sizes. Similarly, poultry genetic resources used in research are being lost, as experimental lines of chickens developed to investigate a multitude of traits are being eliminated by research and governmental institutes (Fulton and Delany, 2003). Smaller-scale efforts to cryopreserve rare breeds could be combined with efforts being made in commercial breeding in order to safeguard genetic resources on both a national and global scale (Blesbois et al., 2007). It is noteworthy that cryopreservation programs could be financially viable for poultry flocks after as little as 3 years and using conventional backcrosses to re-establish the genome of the poultry breed (Silversides et al., 2012).

As it remains experimentally unachievable to cryopreserve avian oocytes or early-stage embryos, an entire chicken breed cannot be reconstituted using conventional cryopreservation technologies (Petitte, 2006). The cryopreservation of chicken male gametes can be achieved using traditional methods of semen preservation. There are inherent problems with using semen for reconstitution of chicken breeds (Blesbois et al., 2007). Semen viability after cryopreservation has proven variable between poultry breeds, and as the female chicken is the heterogametic sex containing the W sex chromosome, the entire avian genome cannot be conserved using semen preservation. Therefore, frozen semen collections can only be effectively used to safeguard and increase the genetic diversity of extant chicken breeds. An alternative method for the cryopreservation of avian gametes is the cryopreservation of gonadal tissue followed by organ transplantation into host chickens. The frozen gonad (testicular or ovarian tissue) is transplanted into immunocompromized hosts and can produce functional semen and oocytes (Song and Silversides, 2007a,b). However, this procedure has not yet produced pure-bred offspring from the direct mating of a male host carrying donor testis tissue with a female host carrying donor ovary tissue.

The use of early germ cell precursors, the PGCs in avian species offers an innovative platform to reconstitute chicken breeds from frozen materials. The PGCs are formed very precociously during avian development. It has been demonstrated that these cells can be isolated from the embryonic circulatory system. The cells can be reintroduced into the circulatory system of host embryos and will colonize the host gonad and produce viable male and female gametes in hosts of the same sex (Naito et al., 1994; Song et al., 2005; Nakamura et al., 2010a; Nakamura et al., 2012). The difficulties associated with this method are that only 100 to 200 PGCs in total are present in the circulatory system of the embryo at this stage and the exogenous introduced PGCs must compete with the endogenous PGCs present in the surrogate host embryo. Nevertheless, using this technique, a pure rare-breed chicken was reconstituted from frozen PGCs using surrogate hosts of common chicken variety (Nakamura et al., 2010b).

**Figure 1.**
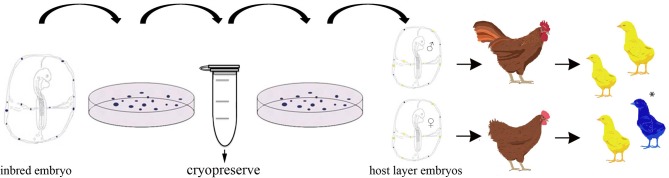
Diagram of a PGC biobank and reconstitution of offspring from cryopreserved PGCs. Blood is isolated from a single embryo and the PGCs are cultured until reaching more than 100,000 cells. The PGCs are then frozen in multiple aliquots. At a later time, a vial of PGCs is thawed and cultured for several days to allow the PGCs to recover. The PGCs are injected into surrogate host embryos and then incubated until hatching. The hatched chicks are sexed and the chicks containing injected donor PGCs of the same sex are raised to sexual maturity. The surrogate hosts are crossed in test matings. Some of the resulting offspring will inherit their genome from the donor PGCs, indicated by an *.

The development of in vitro culture conditions for expanding the population of PGCs before cryopreservation potentiates the ability to safely store cells before transplantation and reduces the technical skill needed to isolate and purify the cells before transplantation. A schematic showing the experimental steps in this protocol is shown in Figure 1. Germ line transmission has been shown from many laboratories using high serum medium conditions (van de Lavoir et al., 2006; Choi et al., 2010; Macdonald et al., 2010; Macdonald et al., 2012; Miyahara et al., 2014; Song et al., 2014; Naito et al., 2015). Recently, we developed a defined medium for the culture of chicken PGCs (Whyte et al., 2015). Defined serum conditions should allow for the standardized culture of PGCs that can subsequently be transferred between research institutes.

As a first step in demonstrating the biobanking of chicken breeds using in vitro cultured and cryopreserved PGCs we have used 5 highly inbred White Leghorn lines of chicken currently maintained at the National Avian Research Facility (NARF), UK. These White Leghorn lines were back crossed and selected to express a single MHC haplotype and vary in their susceptibility to many avian viral and bacterial pathogens The lines used in this study are the inbred line 6 (varying in their susceptibility to Marek's virus) the partially inbred line O lacking endogenous avian leucosis viruses (ALVs), Cornell partially inbred lines N and P (differing in resistance to MD), and the Wellcome inbred line W; (lines 6, O, N, P, W) (Cole, 1968; Bacon et al., 2000). These chicken lines have been maintained for several decades as breeding populations in several countries and have been used in vaccine development and the identification of genes involved in disease resistance to several pathogens. Although partially inbred, these lines still contain some genetic variability which is much reduced compared to outbred lines (Gheyas et al., 2015). The use of inbred lines also demonstrates the capacity to biobank low-fertility, specialty breeds using cultured PGCs.

Here we describe the establishment of a biobank of frozen primordial germ cells from 5 chicken lines of research interest. Each archived line comprises between 15 to 32 individuals in total, and at least 5 individuals of each sex.

## MATERIALS AND METHODS

### PGC Culture Medium

Avian PGC culture medium contained 1 × B-27 supplement, 2.0 mM GlutaMax, 1 × NEAA, 0.1 mM β-mercaptoethanol, 1 × nucleosides, 1.2 mM pyruvate, 0.2% ovalbumin (Sigma), 0.2% sodium heparin (Sigma) 5 μg/mL in avian DMEM, a custom basal medium (a modification of knockout DMEM [250 mosmol/L, 12.0 mM glucose, and CaCl-free; ThermoFisher Scientific]). The following growth factors were added before use: human Activin A, 25 ng/mL (Peprotech); human FGF2, 4 ng/mL (R&D Biosystems); 0.2% chicken serum (Biosera). All reagents were purchased from ThermoFisher Scientific unless otherwise specified.

### Chicken Lines, PGC Line Derivations and Embryo Manipulations

Chicken flocks of several partially inbred White Leghorn lines were produced and maintained at IAH, Compton and are now housed at the NARF, UK. Line 0, 6 and N birds originate from the Poultry Research Laboratory, East Lansing, MI. Lines N and P line birds from Cornell University, USA and Wellcome B14 line (W line) from Wellcome Research Laboratories, Beckenham, UK.

Inbred PGC lines were derived by placing ∼1.0 μL of blood isolated from stage 15 to 16 (H&H) embryos in 300 μL medium in a 48-well plate. One-third of the medium was changed every 2 d. When total cell number reached 1.0 × 10^5^, total volume of medium was changed every 2 d and cells were propagated at 2 to 4 × 10^5^ cells/mL medium. Cells were frozen in Avian DMEM containing 4% DMSO/5% chicken serum and stored at –150°C. The donor embryo was isolated and sex was determined as published (Macdonald et al., 2010).

To investigate the efficiency of germline transmission, one male and one female line 6 cell line were expanded in culture in FAcs medium and cryopreserved for 1 wk. Cells were thawed, cultured for several wk and counted. Eggs were incubated until stage 16 HH and windowed through the pointy end. 5,000 to 6,000 cells total of male or female cells were injected into the dorsal aorta, the egg was resealed with parafilm and incubated with the pointy end down until hatching (MacDonald et al., 2010; Park and Han, 2012). The hatched chicks were raised to sexual maturity. Genomic DNA extracted from semen of the adult chimeric roosters was initially screened in a PCR reaction using the lei 221 primers to identify the presence of the 190 bp allele in the semen (McGrew et al., 2004). One surrogate host rooster was crossed to wildtype hens and pooled semen from wildtype roosters was used to inseminate the surrogate host hens. CAM (chorio-allantoic membrane) and blood samples were collected from the hatchlings. Genomic DNA was prepared using the QIAamp DNA micro kit (Qiagen). Genomic DNA was then subjected to microsatellite analysis to identify donor PGC-derived offspring. Animal experiments were conducted under U.K. Home Office license.

### Microsatellite Analysis

Microsatellite analysis was performed by Source Bioscience using the following primers: Lei 221: CCTTTATCCACTCTTCATGCAC; TGCATAAATTCCATGGGTAAGC Lei 258: CACGCAGCAGAACTTGGTAAGG; AGCTGTGCTCAGTCCTCAGTGC MCW 145: ACTTTATTCTCCAAATTTGGCT; AAACACAATGGCAACGGAAC. PCR products were analyzed using an ABI Prism 3730 Genetic Analyzer.

## RESULTS

### Derivation and Cryopreservation of Male and Female PGC Lines

As a test case, we used eggs from several lines of inbred chicken currently maintained at the National Avian Research Facility, UK. These lines were backcrossed for several generations to generate lines with single MHC haplotypes. The fertility in these lines has been reported to vary between 25 to 75% hatching rate.

**Figure 2.**
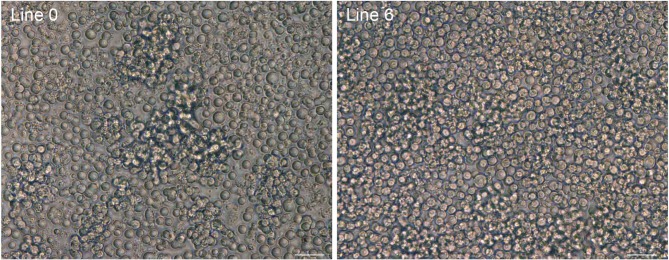
PGCs cultured from inbred chicken lines. An example of derived PGCs culture from single embryos of Line 0 and line 6 chicken lines. Bar, 50 μm.

Fertile eggs were incubated for 2.5 d (stage 16 Hamburger & Hamilton (HH)) and 1 μL of blood was aspirated from the dorsal aorta and transferred to culture medium. The blood sample was cultured in suspension for 2 to 3 wk. During this period the blood cells present in the sample lysed and PGCs present in the well proliferated as single dispersed cells (Figure 2). At the end of 3 wk, the cultures containing more than 100,000 cells were scored as a positive derivation of a cell line, and these cells were cultured for an additional wk in increasing volume (100,000 cells per 0.5 mL medium) and then frozen in aliquots of 50,000 to 100,000 cells per vial.

The data in Table 1 show the results from these experiments using 5 lines of inbred chicken. A total of 203 cultures were started from single embryos. Approximately 630 eggs were incubated to obtain this number. This 33% initiation rate was due to 1) infertility of the incubated eggs, 2) loss of fertility during shipment and storage of the eggs, 3) developmental abnormalities from the inbred lines, and 4) variation in developmental stages between lines which resulted in embryos that were too young (less than stage 15^+^) or too old (older than stage 17 HH) for blood sampling.

**Table 1.** Generation of frozen single-genotype, sexed PGC lines from lines of specialized inbred chicken.

In-bred	No. of	Male PGC	Male PGC	Male PGC	Total	Female PGC	Female	Female PGC	Total	Total	Total	Total	Total
line	expts	cultures	geno-types	deri-vation	male	cultures	geno-types	deri-vation	female	cultures	frozen	deri-vation	vials
		initiated	frozen	rates (%)	vials	initiated	frozen	rates (%)	vials	initiated	geno-types	rates (%)	
6	4	15	12	80	45	18	15	83	52	33	27	82	97
N	5	32	13	41	52	27	16	59	62	59	29	49	114
O	3	17	13	76	52	25	17	68	68	42	30	71	120
P	3	19	18	95	58	24	14	58	44	43	32	74	102
W	2	9	5	56	15	17	10	59	30	26	15	58	45
													
All lines		92	61		222	111	72		256	203	133		478

Summary of PGC line derivation results from multiple culture experiments and the total number of cryopreserved samples are indicated.

For 203 cultures initiated from single embryos, 133 independent cell lines (genotypes) were expanded and frozen in a total of 478 vials. The overall derivation rate was 67%, which is consistent with past cell line derivation results (Whyte et al., 2015). The derivation rates for male and female PGC cultures were similar (70% versus 65%, respectively) demonstrating that both male and female genotypes can be captured.

To avoid inbreeding depression, it has been estimated that a minimum population containing 13 breeding pairs is needed (FAO, 1998). We have obtained this number of genotypes for line 6 (27 genotypes), line N (29 genotypes), line O (30 genotypes) and line P (32 genotypes). Line W comprises 15 genotypes and would need further cryopreserved cultures to regenerate an outbred population. Since these chicken lines were partially inbred during the selection for single MHC haplotypes, there is less of a requirement for retaining a higher level of genetic diversity in the stored germ cell lines.

### Germline Transmission of Inbred PGCs through Outbred Surrogate Hosts

To test that the cryopreserved PGC lines were germline competent and as a first step to demonstrate the reconstitution of a chicken breed from frozen propagated PGC lines, we wanted to verify that the cryopreserved PGCs could produce viable gametes when transplanted into an outbred commercial layer line. A male and a female line 6 PGC cryovial was thawed and expanded in culture for several wk. Five thousand to 6,000 male or female PGCs were injected into the dorsal aorta of stage 16 host ISA brown embryos. The embryos were incubated until hatching, hatched, and the hatched chicks were sexed by PCR. The hosts containing correctly sex-matched donor PGCs were raised to sexual maturity. Three hens and 3 cockerels containing donor line 6 PGCs were produced. An initial examination of the cockerel semen using microsatellite analysis tentatively identified the male containing the highest contribution of donor PGCs in the semen (used in Table 3 below). This cockerel and 2 females were crossed to wild-type ISA brown chickens. The mating data indicates that fertility was normal for these surrogate hosts (Table 2). The offspring from these matings were genotyped using microsatellite analysis to determine if they derived from a Line 6 gamete (Table 3). Three[Table tbl3a] individual microsatellite primer pairs were used for this analysis. Primer pair MCW 145 produces a PCR product of 190 bp for line 6 genomic DNA. Primer pair Lei 258 produces a PCR product of 265 bp for line 6 genomic DNA. Finally, primer pair Lei 221 produces a PCR product of either 207/208 bp or 235 bp as the line 6 birds contain 2 different alleles at this locus. Table 3 presents the results of this breeding experiment. Line 6 control genomic DNA from 2 male and 2 female PGC lines (M1, M2, F1 and F2, respectively) clearly produced the predicted PCR products for all 3 primer sets. Genomic DNA from the ISA brown host line did not produce a similar product for primer set Lei221 or Lei 258 but several offspring did contain a 190 bp product for the MCW145 primer set, indicating that this primer set is not conclusive for identifying line 6 offspring from this mating. This is apparent for the offspring from hen 11-12 which contained a band of 190 bp but none of the other microsatellite markers indicative of line 6 offspring (0 for 11 chicks). In contrast, 4 offspring from hen 14-7 (4 of 5 chicks) contained all 3 microsatellite markers indicating that most of the offspring were derived from the introduced PGCs. The surrogate host cockerel was mated to wildtype ISA brown hens and genomic DNA from the offspring was analyzed. One offspring (1 of 45 chicks; 2%) contained all 3 microsatellite markers and was therefore derived from the line 6 donor PGCs. These results indicate that cryopreserved PGCs from an inbred line can produce viable gametes and offspring when transplanted into the embryos of a fertile layer line.

**Table 2.** Fertility of founder birds containing exogenous PGCs.

Founder	Days PGCs	Eggs	Chicks
birds	cultured before		
	injection		
IBL 11–12 **♀**	36	32	21 (65%)
IBL 14–7 ♀	64	19	12 (63%)
IBL 11–6 ♂	36	169	114 (67%)

**Table 3.** Microsatellite analysis of offspring from surrogate host chickens.

		Lei 221		MCW145		Lei 258	
		207/208, 235		190		265	

		Call 1	Call 2	Call 1	Call 2	Call 1	Call 2
	**Donor line 6**						
	**Line 6 PGC ♀**1	**208**	**235**	**190**	**190**	**265**	**265**
	**Line 6 PGC** ♂1	**207**	**208**	**190**	**190**	**265**	**265**
	**Line 6 PGC ♀**2	**207**	**235**	**190**	**190**	**265**	**265**
	**Line 6 PGC** ♂2	**207**	**208**	**190**	**190**	**265**	**265**
	**Host line**						
	**ISA brown ♀**	203	210	**190**	202	310	480
	**ISA brown** ♂**1**	206	214	201	204	308	310
	**ISA brown** ♂**2**	206	214	201	204	308	310
	**Female host 11–12**						
Offspring	**9**	206	210	201	204	249	308
	**10**	210	214	204	204	308	308
	**14**	210	210	201	201	310	310
	**15**	210	210	201	204	249	310
	**18**	210	210	201	201	310	310
	**19**	191	214	**190**	204	249	310
	**20**	210	214	204	204	249	308
	**21**	191	210	**190**	204	249	310
	**24**	210	214	204	204	310	310
	**25**	210	214	204	204	308	310
	**32**	210	210	**190**	201	310	364
	**Female host 14–7**						
Offspring	**9**	**207**	210	**190**	204	**265**	308
	**10**	**207**	210	**190**	204	**265**	308
	**14**	210	210	**190**	204	308	310
	**18**	**207**	223	**190**	204	249	**265**
	**19**	**207**	210	**190**	201	249	**265**
	**Male host 11–6**						
Offspring	**12**	214	214	201	201	310	310
	**13**	210	214	201	204	249	364
	**19**	214	214	201	204	249	364
	**22**	214	214	201	204	310	364
	**25**	214	214	201	204	249	364
	**39**	218	223	201	201	308	310
	**41**	210	218	201	204	249	310
	**53**	214	218	201	201	249	364
	**54**	210	218	201	201	249	249
	**66**	210	218	201	201	249	310
	**79**	214	218	201	204	249	308
	**94**	210	214	201	201	249	249
	**95**	214	214	201	201	249	308
	**96**	210	214	201	201	249	249
	**100**	214	218	201	204	310	364
	**102**	203	218	201	204	310	323
	**112**	214	218	201	201	249	364
	**113**	210	214	201	204	249	310
	**114**	210	214	201	201	249	249
	**117**	210	214	201	201	249	249
	**118**	210	218	201	201	310	310
	**120**	203	214	201	201	249	364
	**121**	210	214	201	201	249	310
	**122**	203	214	201	204	310	364
	**123**	203	214	201	204	249	323
	**124**	203	214	201	204	310	364
	**126**	203	214	201	204	249	364
	**127**	210	214	201	201	310	310
	**128**	203	214	201	201	308	310
	**130**	203	218	201	204	249	364
	**135**	203	**207**	**190**	204	**265**	364
	**136**	218	223	201	201	249	308
	**138**	210	218	201	201	249	310
	**140**	214	214	201	201	310	364
	**143**	210	214	201	204	310	310
	**146**	214	218	201	204	310	364
	**152**	203	214	201	204	310	323
	**153**	210	218	201	201	249	310
	**155**	210	218	201	201	249	310

**Table 3. tbl3a:** continued

		Lei 221		MCW145		Lei 258	
		207/208, 235		190		265	

		Call 1	Call 2	Call 1	Call 2	Call 1	Call 2
	**158**	214	214	201	201	308	310
	**162**	210	214	201	204	249	364
	**164**	210	214	201	201	249	310
	**165**	214	214	201	201	310	364
	**166**	214	218	201	201	310	364
	**168**	203	218	201	204	249	364

The PCR product size is indicated for genomic DNA from control and offspring from the surrogate host chickens. Two allelic calls were made for each genomic DNA sample and line 6 alleles are indicated in **bold**.

## DISCUSSION

The storage and faithful recovery of breeds of poultry from stored germplasm is needed for the long-term safeguarding and management of poultry genetic resources (Whyte et al., in press). Due to the structure of the laid chicken egg, the storage of ova and early embryos has not been possible so multiple cryopreservation methods have not been possible. Cryopreservation of avian species wholly relies on the use of semen. Semen preservation in chicken is variable between breeds and much more difficult than the routine laboratory procedures used for many mammalian species (Whyte et al., in press). Recently, cryopreservation and transplantation of gonadal tissue from both males and females have been developed (Song and Silversides, 2007a,b). It remains to be seen if this will be a viable method and join semen as a preferred method for breed preservation. The requirement of surgery and immunosuppressants shows that this will be a highly technical procedure with welfare issues. The use of the early precursors to the germ cell lineage, the PGCs, offers an alternative method to safeguard valuable flocks of chickens. It has previously been demonstrated that PGCs can be isolated from the early circulatory system of the embryo or from the embryonic gonad, purified, and cryopreserved. These cells can later be introduced into the circulatory system of chicken embryos and will develop into sperm or oocytes that can produce viable offspring. Here we were able to expand and capture hundreds of genotypes over short (4 wk) culture periods. Our results demonstrate that over the period of a few months sufficient PGC lines could be generated to reconstitute a breeding population for a traditional breed of chicken. A method to increase the throughput of germ-cell line processing is needed if this system is to be applied to commercial pedigree breeds of chicken. This is a prerequisite, as commercial chicken lines comprise several hundreds of genetically diverse individuals for the preservation of genetic resources for sustainability and adaptation for future poultry demands.

The reconstitution of a chicken breed using cryopreserved chicken semen and backcrossing is predicted to take 4 crosses to re-establish 97% of the genome of the original breed (Blesbois et al., 2007). The cost of such a program is predicted to be fiscally equivalent to the costs of maintaining a chicken flock for 3 years (Silversides et al., 2012). The additional costs required for the culture and cryopreservation of PGCs to produce a cell-based biobank could be met if we are able to directly mate the surrogate host males and females to reconstitute a chicken breed in a single cross. In the germline transmission experiments reported here, the male transmission rate was low. Although, using cryopreserved semen, we could have reproduced a pure-bred line from the transmitting female surrogate host (14-7). In our previous work, we obtained 80 to 100% transmission rate from female hosts yet only 10% transmission from the male host indicating that transmission rates using male cultured PGCs are much lower than from females in our culture medium (Whyte et al., 2015). Our results reinforce the need for sterile hosts to reconstitute a pure breed especially from male PGCs. Germ cell ablation can be obtained through chemically induced sterility (Nakamura et al., 2008, 2010a) or genetically engineered sterility by mutation of a gene important for avian germ cell development. It was demonstrated that a pure rare breed of chicken could be produced from isolated cryopreserved PGCs via a surrogate host with depleted germ cells (Nakamura et al., 2010b). Future experiments will either use chemically ablated or genetically ablated host embryos to increase the germ line transmission rates.
